# Augmenting Parenting Programs With the Pause Mobile App: Mixed Methods Evaluation

**DOI:** 10.2196/68807

**Published:** 2025-04-30

**Authors:** Nathan Hodson, Peter Ivor Woods, Stephanie Donohoe, Juan Luque Solano, James Gardner, Manuel Giardino, Michael Sobolev, Domenico Giacco

**Affiliations:** 1 Warwick Medical School University of Warwick Coventry United Kingdom; 2 University of Warwick Coventry United Kingdom; 3 University of Buckingham Buckingham United Kingdom; 4 Coventry and Warwickshire Partnership NHS Trust Coventry United Kingdom; 5 Cedars-Sinai Medical Center Los Angeles, CA United States

**Keywords:** digital microintervention, parenting app, parenting, children’s mental health, local authority

## Abstract

**Background:**

Parenting programs are the recommended treatment for common mental health problems of childhood such as conduct disorder. In the United Kingdom, local authorities have responsibility for providing or commissioning these programs through face-to-face and video call weekly groups and e-learning style asynchronous offerings. However, there has been a shortage of research into the potential of digital resources to augment and enhance parenting groups.

**Objective:**

This pilot study aimed to explore whether providing digital microinterventions in a mobile app (Pause) to augment parenting programs is a feasible strategy. Pause fits into parenting programs and prompts and supports parents to use each week’s new parenting skill at home. Specifically, we want to understand (1) whether parents use Pause, (2) what type of features or tools in Pause are most frequently used for support, and (3) what are the perceived strengths and weaknesses of Pause.

**Methods:**

Pause was provided to parents attending 3 of the most common parenting programs delivered across 3 local authorities in the United Kingdom. During weekly sessions, parents were supported to add “tools” in the app, which mapped onto the training in their session, for example, distracting their child, setting age-appropriate consequences, and using praise. Preprogram surveys were obtained at the first session. After programs were completed, postprogram surveys were administered to measure app use, gather which tools parents used, and explore the strengths and weaknesses of the app. Participants and practitioners were invited for interviews, where the strengths and weaknesses of augmenting parenting programs with Pause were discussed in more detail.

**Results:**

In total, 53 parents were recruited from groups. A total of 25 of 53 (47%) parents completed postsurveys distributed at their final parenting group session, in keeping with typical rates of attrition in parenting programs. In addition, 7 parents and 3 practitioners agreed to interviews after the program. Most of the parents (23/25, 92%) had used Pause. Other than the journal, used by 17 parents, the most popular tools were the relax tool and praise tool, each used by 10 parents. Survey data revealed specific strengths and weaknesses of the tools in Pause, particularly highlighting that parents wanted Pause to provide more ideas for distraction or relaxation activities. Interviews revealed the challenges parents attending programs face, the range of family members using Pause, and the diverse settings where it was used. Interviews also revealed specific opportunities for improving the user interface and for addressing challenges in the journaling function.

**Conclusions:**

This pilot study found good acceptability and engagement with Pause. Interviews revealed promising evidence, suggesting that Pause may improve family life and aid child behavior change. Future research should evaluate whether adding Pause to parenting programs increases their positive effects on children’s behavior and mental health.

## Introduction

### Background

Parenting programs are recommended as the first-line treatment for common mental health problems of childhood such as disruptive behavior disorders and attention-deficit/hyperactivity disorder [1-3]. In the United Kingdom, such parenting programs are predominantly commissioned or delivered through local government [4]. Provision tends to include groups (whether face-to-face or via video call) and e-learning style asynchronous offerings [4,5]. However, there has been a shortage of research into the potential of digital resources and mobile technology to augment parenting groups and enhance change in parenting style to better match evidence-based approaches [6,7].

Poor parent engagement is an ongoing challenge in these programs, stopping families from receiving the full benefit of programs, so it is important to evaluate means of augmenting programs to maximize engagement and therefore increase impact. One systematic review found that only around half of parents who start a parenting program finish it [8]. A large study of a web-based parenting intervention found that, in practice, as few as 7% of referred parents complete the program [9]. This problem may arise in part due to the didactic delivery of programs, which is not well-suited to all parents [10]. Moreover, education is not always sufficient for behavior change. Insights from behavioral economics can ensure that interventions to encourage health-promoting behaviors achieve their potential [11,12]. For example, social norm insights can help avoid situations, where interventions aimed at reducing harmful behaviors in fact normalize and increase them [13].

Digital microinterventions provide one potential means of promoting parenting style change due to the focus on optimizing engagement. Digital microinterventions involve a trigger situation prompting a decision rule process, which is mediated by an app but situated within a wider therapeutic process [14].

Pause (Pause Ltd) is a digital platform that was developed to provide parents with a selection of digital microinterventions to augment parenting programs [6]. The digital microinterventions in Pause—called “tools”—each corresponds to one of the skills parents are taught during parenting programs, such as praise, time together, or distract. As the parenting program proceeds and new skills are taught, group leaders support parents to add the corresponding tool to their toolkit on Pause [10]. In between group sessions, they can use those tools to help them increase the frequency and quality of their use of that particular skill. For example, parents can use the “distract tool” to help them think about how to distract their child from emotions or from unwanted behavior. The distract tool includes 4 age-appropriate distraction ideas and includes space for parents to add ideas for ways to distract their particular child. Another tool, the relax zone, guides parents through teaching their child grounding activities, which they can use to manage anxiety or overwhelming emotions. Finally, the consequences tool suggests age- and neurodevelopmentally appropriate consequences and guides parents through the process of setting short feasible consequences and getting back on good terms immediately after. The wide range of tools is designed either to be used in the heat of the moment when parents face a challenge or to structure a planned interaction. Previous papers describe the development of the app [6,7].

Best practice software development is iterative and user-centered. It is therefore important to find out whether the approach taken in Pause is acceptable and engaging to parents, whether any tools are not engaging, and what are the strengths and limitations of this approach.

### Aims

This pilot study aimed to explore whether providing digital microinterventions in a mobile app (Pause) to augment parenting programs is a feasible strategy. Specifically, we want to understand (1) whether parents use Pause, (2) what type of features or tools in Pause are most frequently used for support, and (3) what are the strengths and weaknesses of Pause.

## Methods

### Participants

Participants were recruited through 3 local authorities in the United Kingdom: Wiltshire Council, Leicestershire Council, and Buckinghamshire Council. To accurately draw from Pause’s population of interest, inclusion criteria were pragmatic. Included participants were attendees at a parenting program run by a local authority whether or not they had legal parenthood. People younger than 18 years of age were excluded, and people who did not have a mobile phone were unable to participate.

Seven weekly groups were selected for inclusion based on practical considerations at local authorities. All attendees of these groups running between January and June 2024 were invited to participate in the study. Recruitment was conducted directly by parenting practitioners who offered participants QR codes to complete pre- and posttest surveys on Qualtrics (Lake Technology Management, LLC) and to download Pause.

Parenting practitioners’ leading groups received 2 personalized training sessions from researchers, where they were shown the relevant tools in Pause. They also had access to ongoing technical and practical support from the research team throughout the study.

### Procedures

Practitioners’ leading participating groups had 2 training sessions to show them how to add the Pause app to their programs. This training was different for the 3 groups, which were running different programs (Wiltshire Council used a Care for the Family program, Buckinghamshire Council used a Family Links program, and Leicestershire Council used a Triple P program). There was no control group. In the first session, participants were given a chance to download the Pause app, consent to participation, and complete the preintervention surveys. These surveys included demographic information (sex, decade of age, language spoken, and whether they had a degree), the Parenting Sense of Competence Scale (PSOC), Warwick Edinburgh Well-Being Scale, and the Strengths and Difficulties Questionnaire Conduct Subscale (SDQ-c) [15-17]. At every subsequent session, the practitioner advised participants which “tool” from the Pause app would complement the topic of the session. In every respect aside from the use of the app, practitioners completed the programs as usual. At the end of the study, parents received a second survey, which repeated the consent process and the rest of the preintervention survey as well as including questions about their use of the Pause app and their experience. Specifically, participants were asked which tools on the Pause app had been used. For each tool they had used, they were asked how many times it was used, what was good about it, and what could be improved. All parents who completed these surveys were invited to attend a video call interview with a researcher to explain on how Pause fits into their experience of the parenting program. Finally, parenting practitioners who had led groups using Pause were invited to be interviewed. Interview topic guides are included in Multimedia Appendix 1. Children were not directly involved in this study.

### Ethical Considerations

All participants provided informed consent. Participants received a US $13.38 voucher for each survey and a US $26.76 voucher for the interview, up to a maximum of US $53.52. Ethics approval was provided by the Biomedical Sciences Research Ethics Committee at the University of Warwick (BSREC 14/23-24).

### Analysis

#### Demographics

Demographics were described using descriptive statistics, including SDs for continuous measures and percentages for binary measures. The characteristics of those who dropped out were compared with those who completed both surveys using 2-tailed *t* tests and chi-square tests using Stata (version 17; StataCorp LLC).

#### RQ 1: Did Parents Use Pause?

This research question (RQ) was assessed in 2 ways using data from surveys. First, on the basis that there were no systematic differences between those who completed the postintervention survey and those who were lost to follow-up, we assumed that rates of using the Pause app were the same among those who completed the second survey and those who did not. To measure this, we divided the number who used Pause by the number who completed both surveys.

Second, we calculated the worst-case scenario engagement rate by assuming that none of those who failed to complete the second survey downloaded and used Pause. To measure this, we divided the number who used Pause by the number who completed at least 1 survey.

#### RQ 2: Which Tools Did Parents Use Pause to Support?

This RQ was assessed using a survey self-report of which tools within Pause were used. Descriptive statistics were used to present how many people used each tool and how many times. These results were presented in a stacked bar chart constructed using Microsoft Excel (version 365). Practitioner interviews were used to evaluate the validity of these findings.

#### RQ3: What Are the Strengths and Weaknesses of Pause?

This RQ was assessed using thematic analysis based on 3 data sources, free-text data from all postintervention surveys, parent interviews, and practitioner interviews. Surveys were reported using illustrative quotes from each question. Interviews were analyzed using thematic analysis using Taguette qualitative software, conducted following Braun and Clarke’s 6 stages, and within a philosophical framework informed by user-centered design [18-20]. Specifically, following familiarization with the data (stage 1), the researchers generated initial codes (stage 2) and searched for themes (stage 3) while remaining attentive to the context of users, their journey through parenting support, the usability of the technology, and opportunities for further refinement—rather than viewing the app as a finished product that either “worked” or “did not.” Following these stages, the themes were refined (stage 4), defined and named (stage 5), before being written up (stage 6). Within this framework, the researchers distinguished parents’ experience of existing parenting courses from the technology. The technology is added to augment the courses so both are reported because (1) understanding the use of Pause entails a rich understanding of the experience of people in courses and (2) explicitly reporting both reduces the risk of wrongly eliding the course and the app. The resulting coding frame was reported with illustrative quotes.

## Results

### Participants

In total, 53 parents completed preintervention surveys. Among them, 25 completed the postintervention surveys, 3 of whom had failed to complete preintervention surveys.

The 25 of those who completed the postintervention survey included 22 (88%) female participants, and 3 others were male participants. In total, 3 were in their 20s, 15 were in their 30s, 6 were in their 40s, and 1 was 50 years and older of age. None were in their teenage years. Most of them (n=23, 92%) reported that English was the main language they spoke at home. Only 6 (24%) had a bachelor degree or higher. The mean age of the child they were primarily concerned about was 6.7 (range 3-13) years. A total of 20 were from the Wiltshire site, 3 from Buckinghamshire, and 2 from Leicestershire.

The 22 completers had a mean PSOC score of 55.2 (SD 2.09). In total, 8 (37%) parents scored below the cutoff for low confidence of 58 [21]. The 22 completers had a mean SDQ-c score of 5.5 (SD 2.24), and 18 (82%) scored above the cutoff of 4 for abnormal conduct problems [22]. The 22 completers had a mean adjusted Short Warwick Edinburgh Mental Well-Being Scale (SWEMWBS) score of 18.9 (SD 2.96), and 14 (64%) scored below the cutoff for the lowest 15% of the population (cutoff=19.5) [23].

There were no significant differences between the 22 who completed both surveys and the 29 who only completed the baseline surveys with respect to demographics (sex, age, language spoken at home, educational level, and child age) or survey responses (PSOC, SDQ-c, and SWEMWBS). Table 1 compares those who completed both surveys with those who only completed one.

Seven participants agreed to interviews. All had completed baseline measures. A total of 4 were from Wiltshire, 1 from Buckinghamshire, and 2 from Leicestershire. They were all female participants, all spoke English at home, and 2 had bachelor degrees. In total, 1 was in her 20s, 5 were in their 30s, and 1 was in her 40s. The mean age of the child they were primarily concerned about was 7.4 (range 4-13) years. Compared with all participants, interviewees had slightly higher confidence (PSOC=58.4; *t*_51_=0.943; *P*=.35), slightly lower conduct problems (SDQ-c=5.0; *t*_51_=–1.19; *P*=.24), and slightly higher well-being (adjusted SWEMWBS=19.8; *t*_51_=1.14; *P*=.26). None of these differences were significant at the .05 level. In addition, we interviewed 3 parenting practitioners who had delivered the Pause program. They were all female, and all from Wiltshire Council.

**Table 1:** Comparing those who completed only the preintervention survey with those who completed pre- and postintervention surveys.

	Only preintervention survey	Both pre- and postintervention surveys	Statistical tests	
			Statistics	*P* value
PSOC^a^, mean (SD)	55.1 (1.87)	55.2 (2.09)	*t*_49_=–0.044	.96
SDQ-c^b^, mean (SD)	6.14 (1.81)	5.46 (2.24)	*t*_49_=1.21	.23
WEMWBS^c^ (adjusted), mean (SD)	18.6 (2.36)	18.9 (2.96)	*t*_49_=–0.454	.65
Decade of age, mean (SD)	3.1 (0.62)	3.2 (0.81)	*t*_49_=–0.62	.54
Child age, mean (SD)	5.9 (2.1)	6.5 (2.5)	*t*_49_=–0.93	.36
Female, n (%)	25 (86)	22 (86)	*χ*^2^_1_=0.0003	.99
Speaks English at home, n (%)	25 (86)	20 (91)	*χ*^2^_1_=0.3	.61
Bachelor degree, n (%)	7 (24)	8 (36)	*χ*^2^_1_=0.9	.34

^a^PSOC: Parenting Sense of Competence Scale.

^b^SDQ-c: Strengths and Difficulties Questionnaire Conduct Subscale.

^c^WEMWBS: Warwick Edinburgh Well-Being Scale.

### RQ 1: Did Parents Use Pause?

In total, 23 of 25 (92%) parents had downloaded and used the Pause app, and 2 (8%) did not. Both cited concerns about personal data, and 1 reported thinking that using an app to support parenting was inappropriate. They were 1 male and 1 female participant, both in their 30s, both spoke English at home, neither had bachelor degrees, and both from the Wiltshire site.

Given the absence of systematic differences between those who did not complete the postintervention survey and those who completed both surveys, we could assume that the app uptake rate of 92% (23/25) held across the whole sample. However, it is also possible that an unmeasured factor linked both engagement and completion of the second survey. A worst-case scenario estimate is that none of the 29 who dropped out used the app so only 23 of 53 (43%) downloaded and used the Pause app.

### RQ 2: Which Skills Did Parents Use Pause to Support?

The core components of the Pause app are the child and parent profiles and the journals; in addition to which, there were 15 digital “tools” available to parents. In total, 20 (87%) parents completed the one-off reflective exercise to create a profile for their children, and 15 (65%) completed the one-off reflective activity to create their own parent profile. Only 12 (52%) reported that this was a positive experience, but 2 said that there were too many questions.

In total, 17 (74%) parents had used the journal. Among them, 6 (26%) only used it once, 7 (30%) used it 2 or 3 times, and 4 (17%) used it more than 4 times. A total of 6 (26%) never used it (3 were unaware of the journal, 1 found it confusing, 1 did not think journaling would be helpful, and 1 did not explain why). Among those who used it, for 6 of 17, their favorite feature was writing down thoughts, and for 6 (26%), it was reading it later and noticing their progress. However, when asked about problems with the journal, 7 (30%) reported that they found journaling time-consuming, 3 (313%) reported that they struggled to put thoughts into words, and 2 (9%) reported that they were worried about privacy.

**Figure 1:**
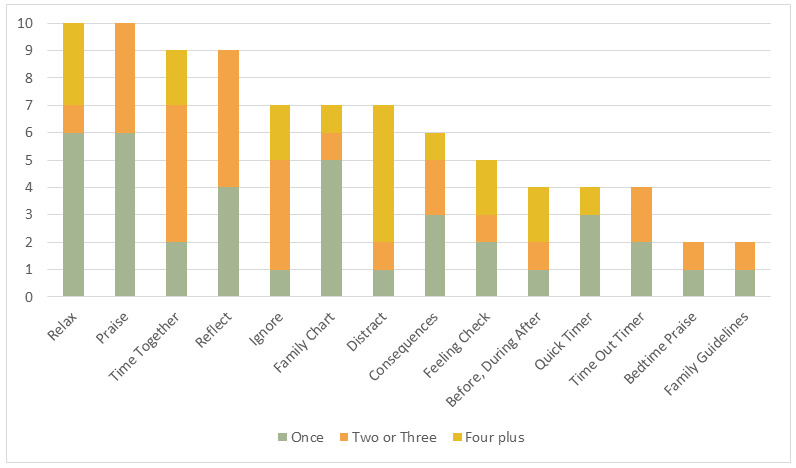
How many parents used each tool and how many times?

In total, 15 digital “tools” were available for parents through the Pause app. Participating parents were in 3 different parenting programs so they were supported to use different tools. The relax and praise tools were used by 10 (40%) parents each. Both the relax and praise tools were used only once by 6 parents, perhaps because they contain learnable reminders. By contrast, the time together and ignore tools were used more than twice by 7 (28%) and 6 (24%) parents, respectively, only 2 or 1 used the tool only once. Similarly, the distract module was only used by 7 (28%) overall, but 5 of those used it 4 or more times. Figure 1 shows how many parents used each tool and how many times.

### RQ 3: What Are the Strengths and Limitations of Pause?

#### Part A: Survey Results

Surveyed parents gave 157 comments about what the “best things” about tools were and also suggested changes. Strengths of the tools included providing a good reminder and a useful distraction, children liking the tools, and creating a helpful structure. Proposed changes included requests for more examples and recommendations, more customization, and simplification. Table 2 describes representative survey results as they relate to each tool.

**Table 2:** Illustrative quotes of best things and suggested changes to Pause tools.

	Best thing	Suggested changes
Relax	“Daughter found it good when she started struggling she would ask for it as said it helped her” and “It created a distraction when my child’s meltdown was about to escalate” [Leicestershire].	“To have more ideas” [Wiltshire].
Praise	“It helps break things down” and “reminder how important it is” [Leicestershire].	“Quite limited, with only a few options” [Wiltshire].
Time together	“It gave us time together and daughter would ask for it” and “The short timers making it easier to show them how long we have” [Leicestershire].	“To add ideas of an activity” [Leicestershire].
Reflect	“Being made aware of how you’re feeling as the parent and how it can affect how we parent, it certainly makes you think about what you can do to change your mood in order to be better for your children and in supporting your children through their emotions and difficulties” [Leicestershire].	“Include ‘sad’ into the emotions” [Leicestershire].
Ignore	“Was a good distraction whilst trying to ignore daughter when she was saying silly/rude words” and “suggestion to distract and timer to help”; “Asks for the reason as to what the need was that caused the behaviour which needed ignoring” [Leicestershire].	“Be able to customise how to ignore not just present one” [Wiltshire].
Family chart	“Being able to set targets for her to follow, and tracking the stars” [Leicestershire].	—^a^
Distract	“Daughter really liked this one, especially if she felt like she was struggling in a situation this would give her something else to focus on and would stop a meltdown” and “Having something to use when out and about” [Leicestershire].	“Add more distract examples” and “be able to customise options” [Wiltshire].
Consequences	“Little games in natural consequences section” [Leicestershire].	“Need to remember to actually look at it. Also didn’t look in the moment” [Leicestershire].
Feeling check	“Making them aware of how they’re feeling at different times” [Leicestershire].	“Suggestions as to what could be making them feel that way” [Leicestershire].
Before, during, and after	“It was a good way to unpick incidents” and “Breaks it down so you can see what happens during a meltdown and what could have triggered it!” [Leicestershire].	“Option to edit which child as I posted to the wrong child and could not change” and “Be able to voice note it would make it easier to document the event” [Leicestershire].
Quick timer	“Handy and the children can see the timer” [Leicestershire].	—
Time-out	“Handy to have easily accessible” [Buckinghamshire].	“Descriptive example of how to use the option” [Wiltshire].
Bedtime praise	“Prompts” [Buckinghamshire].	—
Family guidelines	—	“Long winded” [Wiltshire].
My body	“It gives you prompts on what to discuss with your child when talking about body” [Wiltshire].	—

^a^Not available.

#### Part B: Interview Results

##### Overview

Three themes emerged from these interviews: (1) features of programs without Pause; (2) the interface between parent, child, and Pause app; and (3) the outcomes and effects of the Pause app. The themes are outlined in Table 3.

**Table 3:** Macro- and microlevel themes.

Macrolevel themes	Microlevel themes
Theme 1: features of programs without Pause	Theme 1.1: what made programs effective? Theme 1.2: what made programs difficult?
Theme 2: the interface between parent, child, and app	Theme 2.1: how Pause was used? Theme 2.2: interaction with the app
Theme 3: the outcomes and effects of Pause	Theme 3.1: Pause enhances parenting programs Theme 3.2: Pause improves behavior and enhances family life

##### Theme 1: Features of Programs Without Pause (Overview)

Rather than solely discussing Pause, we also explored parents’ experience of the program in order to ensure that the strengths and weaknesses of programs in general were not elided with the strengths and weaknesses of Pause. We report these below to illustrate the context in which Pause is being used.

##### Theme 1.1: What Made Programs Effective?

Participants talked about how their groups were useful because of program content, other parents, and effective practitioners. Nine commented on the content of the program, always positively.

I really enjoyed it. I found out a lot of helpful information, so it was really good.

Participant 2, parent

The composition of the group, other parents facing similar challenges at home, also added value to the group experience for 4 parents.

I’m going through all the things with my child by myself. It kind of makes you feel isolated, so hearing from other parents that they’re having the same or similar issues, I think that was the most helpful because it made me feel like it wasn’t just me.

Participant 1, parent

The careful and inclusive didactic approach of practitioners was also highlighted as a key feature of what made groups useful. Three practitioners specifically mentioned the quality of the explanations.

they made sure everyone understood each week what we were doing and made sure by the end of it, everyone was clear of what it was.

Participant 5, parent

##### Theme 1.2: What Made Programs Difficult?

The reasons parents were attending groups also made programs difficult. Specifically, family life was already very difficult due to their children’s challenges, and many demands on their time and attention could also lead to attendance problems. Four parents described the problems that had motivated them to seek help for their family, which were primarily due to externalizing behaviors such as meltdowns, anger, violence, and shouting.

My son is quite angry and has a lot of angry outbursts. For a long time, I thought he was just being boisterous, but when the same problems started happening at school, I realized there might be something else going on.

Participant 1, parent

Six participants commented on the children’s neurodevelopmental differences, special educational needs, or subclinical behavior problems, as well as the challenges of long waiting lists and parents’ own problems. They did not mention anxiety or low mood among their children.

Three out of my four (are) diagnosed autistic and ADHD. My 4 year old is ... he’s on waiting lists but he’s not quite being diagnosed yet, but he has got traits. I’ve looked back into my past and things like that and I’m now on waiting lists for an ASD and ADHD diagnosis for myself.

Participant 6, parent

All parents were asked about their attendance. Parents and practitioners identified that it is hard for single parents to attend, especially when they have several children with neurodevelopmental disorders, but parents also reported that events come up. Video call sessions were thought to have made it easier to attend.

It’s rare that you’ll get a parent that will attend every single week, because there may be something that’s come up, you know, like, obviously, the parents are there and the children have got disabilities.

Participant 8, practitioner

All 3 practitioners, but no parents, also gave examples of reasons parents struggle to engage such as circumstances or demographics. For example, unwilling to change parenting style, feeling overwhelmed in groups, or requiring translation.

Another barrier is if parents are not willing to make changes but blame everything onto the child.

Participant 8, practitioner

##### Theme 2: The Interface Between Parent, Child, and App (Overview)

Participants reported how Pause was used, noting which people used it, where they used, and which parts of the app they used. They also commented on their experience of interacting with the app, including the user interface and the idea databases.

##### Theme 2.1: How Pause Was Used

Participants commented on who, where, and when Pause was used and which parts of Pause were used. Nine participants mentioned who used Pause. Pause was used by parents and children. Sometimes it was used by parents and children together, sometimes by parents without their children, and occasionally by children alone. Sometimes it was only used with 1 child, sometimes it was used with all children, and sometimes it was used by the extended family.

I’ve got two children. The oldest is nearly 15, so he wasn’t really involved that much. But the youngest is 4, and it was very age-appropriate.

Participant 1, parent

Five participants mentioned the settings or scenarios where Pause was used. Parents used the app at relatives’ houses, in shops and restaurants, and in their bedrooms alone. They used it during the day, with their children, and also at night alone.

It’s that big time reassurance that we’re going out. We’ve just been to my nan’s and we’ve had meltdown after meltdown. So I said to her, do you want to do the breathing? And she said yeah, and she sat there with it for a good five minutes, like going through them all. So yeah, it’s a great reassurance knowing it’s just there and it’s a click of a button away.

Participant 5, parent

Eight participants commented on specific modules. In total, 11 different modules were mentioned as particularly useful, particularly relax and distract, which were mentioned favorably by 3 parents.

We also used the “distract” function a lot. If something was brewing, I’d quickly check for an idea, and now I have a few ideas in my head. It’s hard to think on the spot when everything is going on, so it was helpful to have ideas that worked, and it could shift their thought process for a minute.

Participant 3, parent

Five participants commented specifically on the function of Pause as a reminder. Parents appreciate Pause providing reminders of when and how to use evidence-based skills.

it’s been quite helpful as, like, reminders to do things. I’ve always felt like I’m quite good at praise, but actually having that reminder there, because things can get quite negative quite quickly when you’ve got behaviors that are quite unwanted. But it’s quite good as a reminder.

Participant 7, parent

Five participants commented on the good fit between the modules of the Pause app and the content of the parenting programs.

I liked, as the course was going on, you could add the modules that you were talking about.

Or, you know, so you didn’t have everything all in one go. So, as you were going on in the course, they were like, “oh, you can add this module now to your app.” I liked that feature because then ... You haven’t got everything in one go.

Participant 6, parent

##### Theme 2.2: Interaction With the App

Participants commented freely on the strengths and weaknesses of the user interface, offering ideas for additional features of Pause and particularly requesting additional personalized ideas added to the app.

Nine participants commented on the user interface. Overall, and in general, parents found it easy to use the app. When assigned tasks during the interview, they were almost always able to find things immediately, and all succeeded in the tasks. There were instances of bugs where text entry boxes and buttons did not display properly because the app remains in its infancy.

It’s really easy to use. So it’s fairly self-explanatory. So as you go on to it, you’ve got your homepage with different sections. Obviously, you can add the sections you want. And it has come in really handy.

Participant 5, parent

Parents engaged with the app and had suggestions about potential features that could be added to the app or ideas for improvements to the app. Seven participants offered ideas for additional features. These suggestions related to both interaction with the app and the functions of the app.

I wish there was a branch-off for a child version, so he could have it on his iPad. Then he would know when he’s starting to feel frustrated. I’ve implemented a “busy bag” for him, so he might say, “Mum, I think I need 5 minutes quiet time, I’m going to take my busy bag.” So maybe there could be a child version of the Pause app, where he could blow out the candles on his iPad or something like that.

Participant 4, parent

Is there a thing in the app where they can request help or ask questions if they’re not sure about something?

Participant 8, practitioner

Five participants indicated that Pause was particularly useful in providing a database of ideas, which added value for parents.

If he’s escalating, I can use the app to prompt him, like “How many colors can you name?” I’ve even sent him to quiet time with a task like, “Can you name as many animals as you can think of?” He’s only there for 3 minutes, but it helps.

Participant 4, parent

Journaling is a central component of the app so parents were specifically asked about any difficulties with reflection. Several people found reflection hard despite the prompts in Pause, and there is more work to do to ensure everybody gets to reflect on their parenting using Pause.

It’s just sometimes finding the time to go on your phone to do those things. A lot of the time, things are going on in my head, if you know what I mean, but aren’t necessarily on paper or on my phone.

Participant 7, parent

##### Theme 3: The Outcomes and Effects of Pause (Overview)

Parents and practitioners reported that Pause was useful during parenting programs, and parents continued to use it after their programs had finished. Parents reported that Pause had improved their children’s behavior and their family life as a whole, linking this to the convenience of using an app. They also reported improved insight into their child’s behavior and their own behavior, linking this to the reflection encouraged in Pause.

##### Theme 3.1: Pause Enhances Parenting Programs

All participants reported that the app helped them. Describing it as “brilliant” (Participant 4, parent) or “really useful” (Participant 2, parent) and explaining that they use it “regularly” (Participant 3, parent).

I would say life-changing, really, because before, in a blink of an eye, she could run. And she’s fast. Now she will ask for it and it will calm her. So, it has for us made it slightly better going out. Even going to a restaurant now, she will sit with it and she will get the app up and get it up herself. If she’s doing the five senses one, she’ll walk around the restaurant looking for different stuff and smells. So it has changed a lot for us and it has really helped.

Participant 5, parent

Seven participants noted that the app remains useful after the sessions have finished, including all 3 practitioners.

I really liked it, and I still use it now. Even though the course has finished, I still use it.

Participant 2, parent

Once they’ve done the program and they’ve got the app, they’ve got it forever. So there’d be no need to come back through the program.

Participant 10, practitioner

##### Theme 3.2: Pause Improves Behavior and Enhances Family Life

Three parents attributed improvements in their child’s behavior to Pause.

We’re out and about and then we feel it’s coming, it just, I mean, it works for minutes with her. Once she starts doing it, she’s like, I’m calm.

Participant 5, parent

Now, he’s starting to tell me, “I feel a bit fidgety, I need to do something.” It would help him take control and gain more independence in regulating himself.

Participant 4, parent

Four attributed improvements in their family life to Pause. Parents described how Pause improved life for the whole family, the child who was referred, plus other children and parents.

Having the use of the Pause app, where I’ve been able to take things like consequences and reward systems with me easily, rather than carrying a jar of buttons or a sticker chart—it’s enabled me to put other things in place to make life a bit more manageable. Like, when I know there’s going to be a meltdown, I can use techniques like “blow out your candles” to stop things before they escalate. So yeah, it’s been a really, really good learning curve, and it’s been brilliant to be able to apply it to all my children, not just the one who was referred.

Participant 4, parent

Five participants commented that the effectiveness of Pause came from it being a mobile app. Specifically, Pause is with parents in their pockets all the time wherever they need it, and parents and children enjoy the interactive nature of sharing the app.

They found it useful to be able to refer back to it, because obviously their mobiles are around all the time rather than have, you know, like paperwork.

Participant 8, practitioner

Six participants commented on the improvements in insight into children’s behavior and what drives them.

She said, I’m so glad I’ve got a Pause app because I used it the whole time through halftime when I felt like I was just losing the plot. And she kept saying that she kept going back to the pause app and it really helped to support her child to know like how he was feeling and to be able to do the before, during and after so she could pick up the patterns and work out why her child was finding, I don’t know, doing the particular activity or anything challenging.

Participant 10, practitioner

Four participants commented on how parents had also gained insights into their own behavior.

It’s about taking a step back. We don’t need to shout and yell; we can deal with it calmly. It’s about making people aware of how to deal with that child and what works.

Participant 4, parent

Six participants suggested that reflection had improved due to the Pause app.

Especially when I was doing the “before, during, and after,” I’d do that on my own and write it down, which I liked because it made me realize, “actually, this happened before,” whereas previously, I wouldn’t pay attention to that. You’d just think, “what’s going on now?” But looking back, I could see it was a trigger.

Participant 3, parent

## Discussion

### Summary of the Results

This pilot study aimed to explore whether providing digital microinterventions to augment parenting programs via the Pause app was feasible. We explored (1) whether parents used Pause, (2) which tools parents used, and (3) what the strengths and weaknesses of the app were. We addressed questions (1) and (2) using a survey of parents who attended groups where Pause was offered. We addressed question (3) by combining survey data with interviews. In total, 25 of 53 (47%) parents completed postsurveys. Only 7 parents and 3 practitioners completed interviews. A total of 23 of 53 (43%) parents who completed presurveys had downloaded and used the Pause app (ie, 92% of those who completed postsurveys used the Pause app). Other than the journal, used by 17 parents, the most popular tools were the relax tool and praise tool, each used by 10 parents. The survey data revealed specific strengths and weaknesses of the tools in the Pause app. Interviews revealed the challenges existing in programs without Pause, the way Pause was used by many family members in diverse settings, and how Pause enhanced parenting programs and improved outcomes for families. Interviews also revealed specific opportunities for improving the user interface and for addressing challenges in the journaling function.

### Comparison With the Literature

The main digital alternatives to parenting digital microinterventions are e-learning programs. This study has shown that Pause can offer a different approach. The key limitations of e-learning programs are “providing a ‘one size fits all’ program with no adaptation or tailoring to the user’s state” and “lack of referrals to in-person treatment in relevant cases” [24]. The biggest studies indicate that only around 7% of referred parents complete these interventions [9]. By using Pause to enhance, rather than replace, parenting groups, we ensured that parents received the benefits of tailoring and personalization, which come from group work with a facilitator. This approach is in line with a growing body of research in digital mental health, which highlights the need for human support to ensure long-term engagement and effectiveness of digital tools [25].

In this study, we estimated that 92% of parents used the Pause app sometimes, but acknowledged that it could be as low as 43%, if none of the survey nonresponders downloaded the app. Either way, this figure is difficult to compare with e-learning style programs where sessions are concurrent and so a completion rate can be calculated [26-28]. Patchy engagement is the norm in digital mental health interventions, and the most important factor is whether parents use evidence-based skills at the appropriate time or whether they do not. Nevertheless, the results of this study indicate that further research to evaluate the effect of the app on parent and child outcomes is appropriate.

### Strengths and Limitations

This study tested Pause in a natural setting rather than a laboratory or staged parenting program; this evaluated both the practitioners’ engagement with distributing Pause themselves and parents’ engagement with the app within the context of the parenting program. Similarly, there were no clinical exclusion criteria so the participants were representative of parenting group attendees in the United Kingdom.

Retention was good within the context of parenting programs. We obtained postprogram surveys from 25 of 53 (47%) parents. Systematic review evidence suggests that 51% of parents disengage before or during long parenting programs such as these, so the proportion of postprogram surveys obtained is likely to represent a large proportion of retained parents [8]. Moreover, 2 parents who did not use Pause completed postprogram surveys, reassuring us that those who did not engage with the intervention were not excluded from data collection. In addition, even after inevitable attrition, the number of included parents was typical for a pilot study of a digital parenting intervention [29-32].

Finally, this paper was strengthened by the use of 4 sources of data: pre- and postprogram surveys, interviews with parents, and interviews with practitioners. These different forms of data collection allowed a rich understanding of how Pause was used within groups.

The main limitation of this study was the self-reported use data. We asked parents to refer to their app to report which “tools” they had added on the app, but it is possible that errors could be made in this process and social desirability bias could have an effect. Although our study design did not include a formal analysis of how frequently tools were used, the self-report data are in keeping with the app feedback, providing some reassurance regarding this potential limitation.

### Implications for Research and Practice

Altogether, this pilot study provides preliminary evidence that Pause can be incorporated into parenting programs with positive effects for many parents and no evidence of adverse effects. These findings suggest that it is appropriate to continue honing the Pause app and exploring its short-term and long-term effects on parenting style. Future research should explore whether incorporating Pause into parenting programs leads to greater change in child behavior outcomes such as the Strengths and Difficulties Questionnaire compared with treatment as usual [17]. We used Pause alongside 3 parenting programs in this study, Triple P Primary, Family Links Nurture, and Care for the Family’s Time Out for Parents. Future research should explore whether the results are the same with other programs.

In their paper, outlining the basis of digital microinterventions, Baumel et al [14] imagined a “hub” bringing together a suite of digital microinterventions. Pause provides that function for the suite of “tools” in the app. They outlined three areas for research: (1) microrandomized trials, (2) optimizing microintervention suites, and (3) understanding for whom digital microintervention care is suitable for. This paper shines light on (2) and (3) by interrogating the strengths and weaknesses of these microinterventions (allowing optimization) and revealing who uses them (at least 43% of parents who start programs in the included UK local authorities). It also provides proof of concept for augmenting parenting programs with mobile technology, similar to other research in digital mental health [33,34]. We plan to further contribute to research on microinterventions in parenting by exploring options to use microrandomized trials for engagement with Pause [35,36].

### Conclusions

The Pause app can be combined with commonly used parenting programs and is well-received by parents. Parents and practitioners identified that Pause enhanced programs and could provide lasting support after programs had finished, with the potential to enhance family life and improve child behavior. Further research is required to evaluate the effect of adding Pause above and beyond treatment as usual, but results indicate that adding digital microinterventions alongside a face-to-face parenting program is a promising, feasible, and acceptable approach at least for some of the parents.

## Appendix

Multimedia Appendix 1 Topic guides.

## Abbreviations

PSOC: Parenting Sense of Competence Scale
RQ: research question
SDQ-c: Strengths and Difficulties Questionnaire Conduct Subscale
SWEMWBS: Short Warwick Edinburgh Mental Well-Being Scale
