# Supplementary material for: Augmenting Parenting Programs With the Pause Mobile App: Mixed Methods Evaluation
Source: JMIR Pediatr Parent. 2025 Apr 30;8:e68807. doi: 10.2196/68807 (PMC12079061; doi:10.2196/68807)
Supplement: Multimedia Appendix 1 [file pediatrics_v8i1e68807_app1.docx]

**Topic Guide for Parents**

*Start the recording.*

Today I’m going to interview you about your parenting program and about the pause app, it will take about 30 minutes. First, I’ve got to get your consent – did you complete the consent form on the online survey. (YES)

Good so that applies to this as well – do you have any questions for me about it or are you happy to get started?

How did you get on with your parenting group?

What were you mainly hoping for from it yourself?

Did you manage to make it to group every week, or did you miss some? If so, how come?

Did you use the app? *No go red, yes go green.*

Why not?

What would it have taken for you to download it?

What would you want in an app?

How did it feel being in the program without the app?

Can you download it now? 🡪 skip to *orange*

How did you find it? (in your own words)

Did you use it with your children or away from your children or both?

Which features did you find useful?

What made them useful?

How did these uses change your family life?

Was there anything difficult or bad about using the app?

How did you find journaling?

Did you make profiles for you and your children?

What did your child think about you using the app?

Thanks is there anything else you want to add? You will receive your £20 voucher in the next 24 hours.

*Save recording*

**Topic Guide for Practitioners**

*Start the recording*

Today I’m going to interview you about your parenting program and about the pause app, it will take about 30 minutes. First, I’ve got to get your consent – did you complete the consent form and return it? (YES go purple, NO go to consent form then come back to purple)

OK - do you have any questions for me about it or are you happy to get started?

How long have you been leading parenting groups?

How did you get on with your parenting group this time?

What were the biggest challenges with leading your parenting group?

How was attendance at your parenting group? What do you think made parents attend/miss?

Did you offer the Pause app? *No go red, yes go green.*

Why not?

What would it have taken for you to offer it?

What would you want in an app?

Did you ever wish you had used the app?

Can you download it now? 🡪 skip to *orange*

How did you find the groups with Pause? (in your own words)

Did you add a new module at the end of each session?

Did you review the journal entries and recent use at the start of each session?

Which features did parents find useful?

What made them useful?

How did these uses change your group?

Was there anything difficult or bad about using pause?

Did you keep using Pause in all sessions, or was it mainly at the start?

Was there anything else you would have liked Pause to help with? Even if it doesn’t seem possible. (Explore this a bit – have a conversation)

Is there anything we could do to help parents to use Pause?

Did you download the app yourself?

Thanks is there anything else you want to add? You will receive your £20 voucher in the next 24 hours.

*Save recording*
